# Toxicant Induced Changes on Delayed Fluorescence Decay Kinetics of Cyanobacteria and Green Algae: A Rapid and Sensitive Biotest

**DOI:** 10.1371/journal.pone.0063127

**Published:** 2013-04-30

**Authors:** Franziska Leunert, Hans-Peter Grossart, Volkmar Gerhardt, Werner Eckert

**Affiliations:** 1 Leibniz-Institute of Freshwater Ecology and Inland Fisheries, Stechlin, Germany; 2 Institute of Biochemistry and Biology, Potsdam University, Potsdam, Germany; 3 Department of Physics, Regensburg University, Regensburg, Germany; 4 Israel Oceanographic and Limnological Research, Yigal Allon Kinneret Limnological Laboratory, Migdal, Israel; University of California Davis, United States of America

## Abstract

Algal tests have developed into routine tools for testing toxicity of pollutants in aquatic environments. Meanwhile, in addition to algal growth rates, an increasing number of fluorescence based methods are used for rapid and sensitive toxicity measures. The present study stresses the suitability of delayed fluorescence (DF) as a promising parameter for biotests. DF is based on the recombination fluorescence at the reaction centre of photosystem II, which is emitted only by photosynthetically active cells. We analyzed the effects of three chemicals (3-(3,4-dichlorophenyl)-1,1-dimethylurea (DCMU), 3,5 Dichlorophenol (3,5 DCP) and copper) on the shape of the DF decay kinetics for potential use in phytoplankton toxicity tests. The short incubation tests were done with four phytoplankton species, with special emphasis on the cyanobacterium *Microcystis aeruginosa.* All species exhibited a high sensitivity to DCMU, but cyanobacteria were more affected by copper and less by 3,5 DCP than the tested green algae. Analyses of changes in the DF decay curve in response to the added chemicals indicated the feasibility of the DF decay approach as a rapid and sensitive testing tool.

## Introduction

The introduction of chemicals and pollutants in the aquatic environment by anthropogenic activities has become increasingly significant in the past decades indicating the need for an increased monitoring effort of water quality [1]. Appropriate assessment of water pollution in various habitats requires, in addition to knowledge on types and origins of the pollutants, proper analytical methods and understanding of the transport and fate of the pollutants as well as precise quantification of adverse effects on organisms. Identification and quantification of toxins/pollutants in aquatic ecosystems are often complicated by synergistic effects between two or more non-toxic compounds or by indirect effects of the contaminants [2]. Bioassays, which expose the indicator organisms directly to the target solution could circumvent this analytical maze, and in the presence of a biohazard, would react in a manner that allows proper monitoring. Although such an approach does not allow identifying a given chemical substance, it resembles a pre-screening test for the presence of a pollutant above a potentially harmful threshold [3].

Today, automated biomonitoring systems encompass an array of advanced electronic systems, specifically designed to measure changes in physiological or behavioural responses in fish or invertebrates [4]. Another well-known toxicity test is based on the swimming behaviour of the zooplankter *Daphnia magna* [5].

Algae are particularly suitable for bio-testing because of their high abundance and sensitivity to environmental pollution in aquatic systems [6]. However, standard growth rate measurements in the presence of potential hazards require exposure of up to 72 h and, therefore, unsuitable for rapid responses (e.g. [7]). A much faster alternative to algal growth is the non-invasive measurement of the *in vivo* fluorescence which facilitates monitoring of activity changes by affecting photosystem II (PS II) [8]. For example, pulse amplitude modulation (PAM) of prompt fluorescence (PF) is a well-established method for detecting impact of chemicals on algal activity (e.g. [9]–[12]).

A promising alternative to PF is delayed fluorescence (DF). DF, which was first observed by Strehler and Arnold [13], represents a recombination fluorescence at the reaction centres of PS II that can be measured as a very weak fluorescence signal, when photosynthetically active cells are transferred from light to the dark. For green algae, it has been shown that DF intensity, the integral over DF decay kinetic, represents a sensitive and reliable parameter for toxicity tests [14]–[17]. In addition, different chemicals alter the DF decay kinetics in a characteristic manner that renders its use in algal toxicity tests more efficient. [14], [17]. DF occurs because electrons flow back from the electron transport chain (ETC) to reaction centre P680 until the thylakoid membrane is completely discharged. In contrast to PF with decay times in the range of ns, temporal behaviour of DF is determined by electron holes and the liberation of electrons from the electron traps within the ETC. This liberation, which is a thermally activated process, is relatively slow (up to minutes) compared to PF [18]. The shape of the DF decay kinetic, however, is influenced by the redox states of the sources of holes (redox active tyrosine residue Y_Z_, oxygen evolving complex, and the positive charged inner site of the thylakoid membrane) and sources of electrons (components of the ETC: Q_A_, Q_B_, Plastoquinone (PQ), Cyt_b6/f_, P_700_, Ferredoxin and the negative charged outside of the thylakoid membrane) [19]. While the fast decay of DF depends on the state of P680 [20], the slower decay is influenced by the redox state of components of the ETC and from the S2 and S3 states of the oxygen evolving complex [21].

In this study, we used a quantitative approach based on changes in the shape of the DF decay curves upon exposure to chemicals. We measured toxicity effects on the DF decay in the presence of the herbicide 3-(3,4-dichlorophenyl)-1,1-dimethylurea (DCMU, Lancester Synthesis; UK), 3,5 Dichlorophenol (3,5 DCP) a standard substance in biotests [22], [23] and copper, a common industrial and agricultural pollutant, which has a high potential to affect photosynthetic activity as well as other cell metabolism pathways [24]–[26]. We tested the performance of the DF approach with cyanobacteria: *Microcystis aeruginosa* and *Aphanizomenon flos-aquae*, often representing the dominant primary producers in aquatic systems [27]–[29]. To better generalize our results, we have also used two common green algae: *Desmodesmus subspicatus* and *Scenedesmus obliquus*.

## Materials and Methods

### Culture Conditions for Cyanobacteria and Green Algae Strains

We used two different cyanobacteria species and two green algae species. The green algae *Desmodesmus subspicatus* Hegewald et Schmidt (SAG 86.81), *Scenedesmus obliquus* (Turpin) Kütz. (SAG 276-1) and the cyanobacterium *Aphanizomenon flos-aquae* (L.) Ralfs (SAG 31.87) were obtained from the Culture collection of Algae, Göttingen. Further, we tested two strains of *Microcystis aeruginosa* Kütz. Both strains grow unicellular, but differ in their toxicity. While HUB018 lacks Microcystin-LR, HUBW333 contains a high level of this toxin [30], [31]. Cyanobacteria strains were cultured in Z-medium [32], and green-algae were kept in WC-medium [33]. All strains grew semi-continuously by dilution every third day in 1L clear glass bottles (Schott). Cultures were illuminated for 24 h (Osram Lumilux cool daylight, 55 µmol m^−2^s^−1^) and continuously aerated with sterile filtered air (Sartorius, Midisart 2000) at 21.5±1°C. Chemicals for all media were obtained from Merck.

### Chemical Effects on Delayed Fluorescence (DF)

#### System set up

DF measurements were performed with a custom made DF decay kinetic instrument [16]. In brief, the light-adapted phytoplankton suspension is pumped in a closed cycle, from the culture vessel into a dark emission-cell passing on its way an excitation cell where it is illuminated by a red light LED (55 µmol m^−2 ^s^−1^). The decay of the DF signal (680–750 nm) is measured for 90 s by a Channel-photomultiplier (R1463, Hamamatsu, Japan) in the photon counting mode upon the pump stops. Temperature of the double-jacket excitation-cell was kept at constant 21.5°C via an external temperature controller (Thermo Haake C10).

#### Measurements

Three days ahead of each experiment, fresh growth medium was inoculated with cultures. On the day of the experiment, cultures were diluted with fresh medium (without EDTA, to avoid complexation of ions) to a DF start value between 8000 and 12000 (counts s^−1^), corresponding to ca. 50–70 µg Chl_a_ L^−1^. 50-ml subsamples were exposed to the following chemicals: DCMU, dissolved in Ethanol, Copper (as Cu_2_SO_4_, Merck, dissolved in Aqua dest), and 3,5 Dichlorophenol (3,5 DCP; ABCR GmbH+CoKG, Germany) dissolved in Dimethyl Sulfoxide yielding final concentrations in a range of 10^−10^ to 10^−6 ^M for DCMU, 10^−7^ to 10^−5^ M for copper, and 10^−7^ M –2.5* 10^−5^M for 3,5 DCP. Addition of each solvent to a subsample of the phytoplankton cultures served as the respective controls. Additional concentrations of all chemicals were prepared for the dose-response curves with *M. aeruginosa* HUB018 and *D. subspicatus* to reach the saturation impact on changes in the DF decay kinetic. This was done to determine the concentration in which the half-maximal effect could be measured (EC_50_).

After adding chemicals, samples were incubated for 20 min under irradiation (55 µmol m^−2^s^−1^) with the same light sources as used for algal cultivation. DF decay kinetic of each sample was measured for 90 s in five replicates. In order to compare rapid and possible long-term effects of exposure with the test toxins, DF decay kinetics of *M. aeruginosa* HUB018 and *D. subspicatus* were measured for selected toxin concentrations, which were in the range of the short term experiments, after 24 h of incubation. All experiments were done in triplicates.

#### Analysis of the DF signal

**Figure 1:**
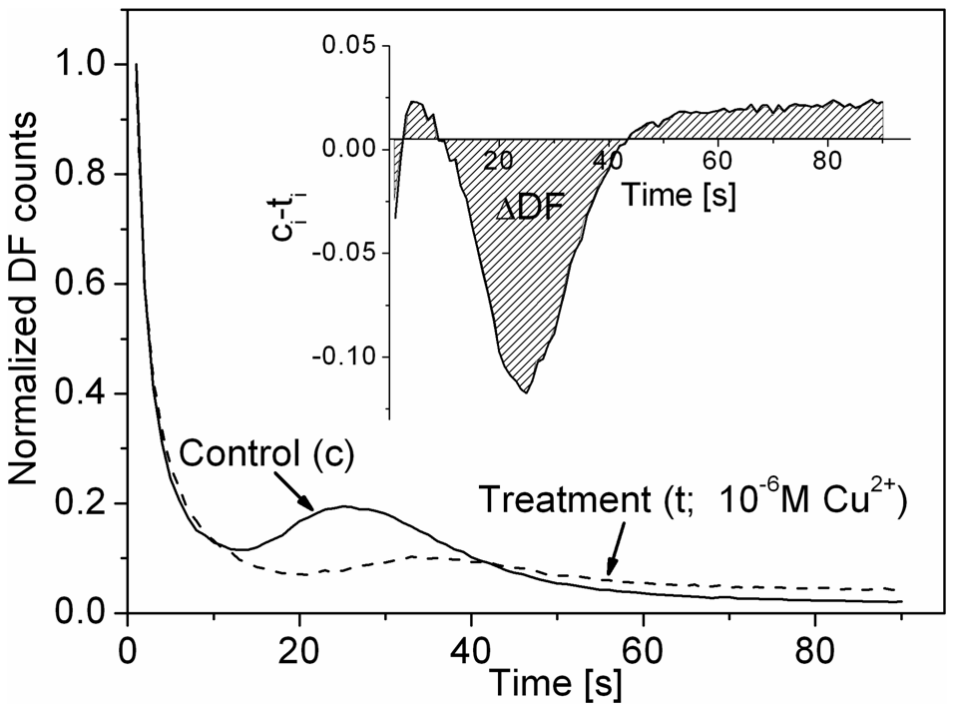
Delayed fluorescence (DF) decay kinetic and its use as a physiological parameter. Normalized DF decay kinetic of the control (black) and after incubation with 10^−6 ^M Cu^2+^ (black, dashed) for *M. aeruginosa* (HUB018); inlet shows residuals of c – t, summed up as ΔDF.

Starting with the control (algal suspension+solvent), each algal sample was measured five times and the last three decay kinetics were averaged for further calculations. To account for possible variability of subsamples and comparability between experiments, the counts of the target sample (t_i_) at each measured time point (incl. those from the control, c_i_) were normalized to the start value (c_1_) of the control. After normalization, the decay curve of the target sample (t in Fig. 1) was subtracted from that of the control leading to the curve of residuals (c_i_ − t_i_) (Fig. 1, inlet). Then, the absolute value of the differences was summed up as ΔDF given in equation 1.
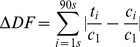
(1)


Accordingly, ΔDF quantifies all changes in the shape of the DF decay curve in comparison to that of the control (Fig. 1). Obtained ΔDF values were compared for the tested phytoplankton species as a function of the concentration of the tested toxin via the dose-response curves.

In addition, ΔDF was related to changes in the DF-integral for *M. aeruginosa* and *D. subspicatus* for short time (30 min) and 24 h incubations. The DF-integral for each DF decay kinetic was calculated using OriginPro8. Hereby, effects on the integral are defined as percent decrease of the integral of the treatment compared to the integral of the respective control. They are given as DFI_30min_ for the short-time tests and DFI_24h_ after 24 h of incubation.

### Growth Inhibition Experiments

Growth inhibition experiments were carried out with non-toxic *M. aeruginosa* HUB018 and *D. subspicatus* to compare the impact of copper, DCMU and 3,5 DCP on growth and DF kinetic. Pre-cultures were grown at 21.5°C in a temperature- and light-controlled incubator (Binder APT. Line KBW), under continuous shaking (90 rpm) using a 16∶8 h light –dark cycle (Osram Lumilux cool daylight, 55 µmol m^−2 ^s^−1^). After algal harvest and dilution in fresh medium, growth inhibition experiments were carried out in the same incubator in 100 ml Erlenmeyer flasks filled with 30 ml culture. Chemicals were added in concentrations from 10^−10^ to 10^−6 ^M for DCMU, copper in the range of 10^−7^ to 10^−4 ^M, and 10^−6^ to 2.5*10^−5 ^M for 3,5 DCP. The solvent alone was added to the control.

As growth parameter, we determined the content of Chl_a_ (mg L^−1^) at the beginning of each experiment and after 1, 2, 3, 6 and 9 days. The standard period of three days (ISO 8692), had to be prolonged because of delay in growth of HUB018. Chl_a_ fluorescence as biomass indicator is a commonly used surrogate parameter for cell number in toxicity tests [34], but several characteristics should be taken into account. Due to temperature and light history dependency as well as dial periodicity [35], [36], [37], it is therefore important to keep this parameters as well as the sampling time constant. For Chl_a_ fluorescence measurement, three 200 µl subsamples of each treatment were transferred into a multi-well microplate (flat bottom, 96-well black fluorescence plates, Nunclon). Measurements were performed with a Synergy 2 microplate reader (Biotek) set as follows: Temperature 27°C, dark adaptation time 40 min, excitation 460/40 nm, emission 680/30 nm. As applied chemicals by themselves influence Chl_a_ measurements, 10 µl of stock solutions were added to the wells using final concentrations of 10^−6^ M DCMU, 2.5*10^−5^ M Cu^2+^ for *M. aeruginosa* HUB018 and 10^−4^ M for *D. subspicatus*. 3,5 DCP had a final concentration of 2.5*10^−5^ M. The Chl_a_ determination via *in vitro* fluorescence in the presence of the substances was calibrated externally with the standard Acetone extraction method according to Parsons and Strickland [38]. Tested chemical compounds were evaluated in four replicates for each concentration, and all experiments were repeated twice.

Growth of the cultures was calculated as increase of Chl_a_ concentration over time. Inhibition was expressed as percent growth of each treatment relative to the respective control (100%) for each measuring time point. To test if DF is a suitable parameter, linear correlation between growth inhibition on days 3 and 9 and the DF parameters ΔDF, as well as the DFI_30min_ and DFI_24h_ for *M. aeruginosa* HUB018 and *D. subspicatus* were calculated.

### Statistical Analysis of the Species Specific Sensitivity

Analysis of species-specific sensitivity at low concentrations was done with linear regression of the four lowest concentrations of each chemical and strain. Slopes of regressions were compared with a t-test and Bonferroni-Holm correction (copper, df = 22, p<0.05; DCMU, df = 22, p<0.05; 3,5 DCP, df = 24, p<0.05).

## Results

**Figure 2:**
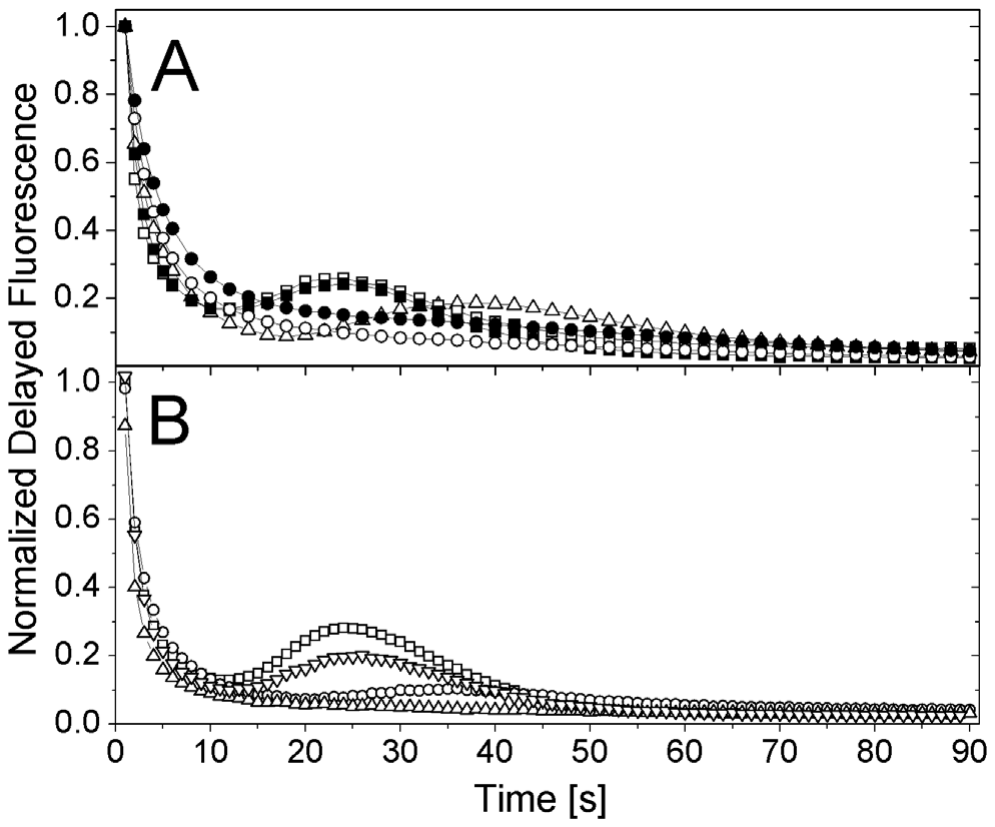
Species specific delayed fluorescence (DF) and effects of toxins. Normalized DF decay curves of **A**: the investigated species, *Aphanizomenon* (open triangle), *Microcystis aeruginosa* HUB018 (filled squares), *Microcystis aeruginosa* HUBW333 (open squares), *Scenedesmus obliquus* (open circle) and *Desmodesmus subspicatus* (filled circle) and **B:** Influence of toxins on DF decay curve of *M. aeruginosa* HUB018; control (open squares), 10^−5 ^M copper (open circles), 10^−7 ^M DCMU (open triangle up), 2.5*10^−5 ^M 3,5 DCP (open triangle down).

The normalized DF decay curves of control suspensions of the five tested phytoplankton species revealed pronounced differences (Fig. 2A). Particularly in cyanobacteria, a transient peak could be observed with variable, species-specific heights and timing. In contrast, decay curves of green algae did not show any peak at any of the experimental conditions applied.

Changes in DF decay kinetics are exemplified in *Microcystis* HUB018 (Fig. 2B), where the control DF decay kinetic showed a clear transient peak with a maximum at 25 s the peak was lower after exposure to the highest concentration of 3,5 DCP (peak height control: 0.3; 3,5 DCP: 0.21). Copper suppressed the transient peak nearly completely and, after DCMU addition, no transient peak occurred. In addition, the DF decay during the first seconds was affected differently. While DCMU caused the strongest decline in DF (DF signal at 1 sec = 0.9; at 2 sec = 0.3), copper and 3,5 DCP did not steepen the initial DF decay.

**Figure 3:**
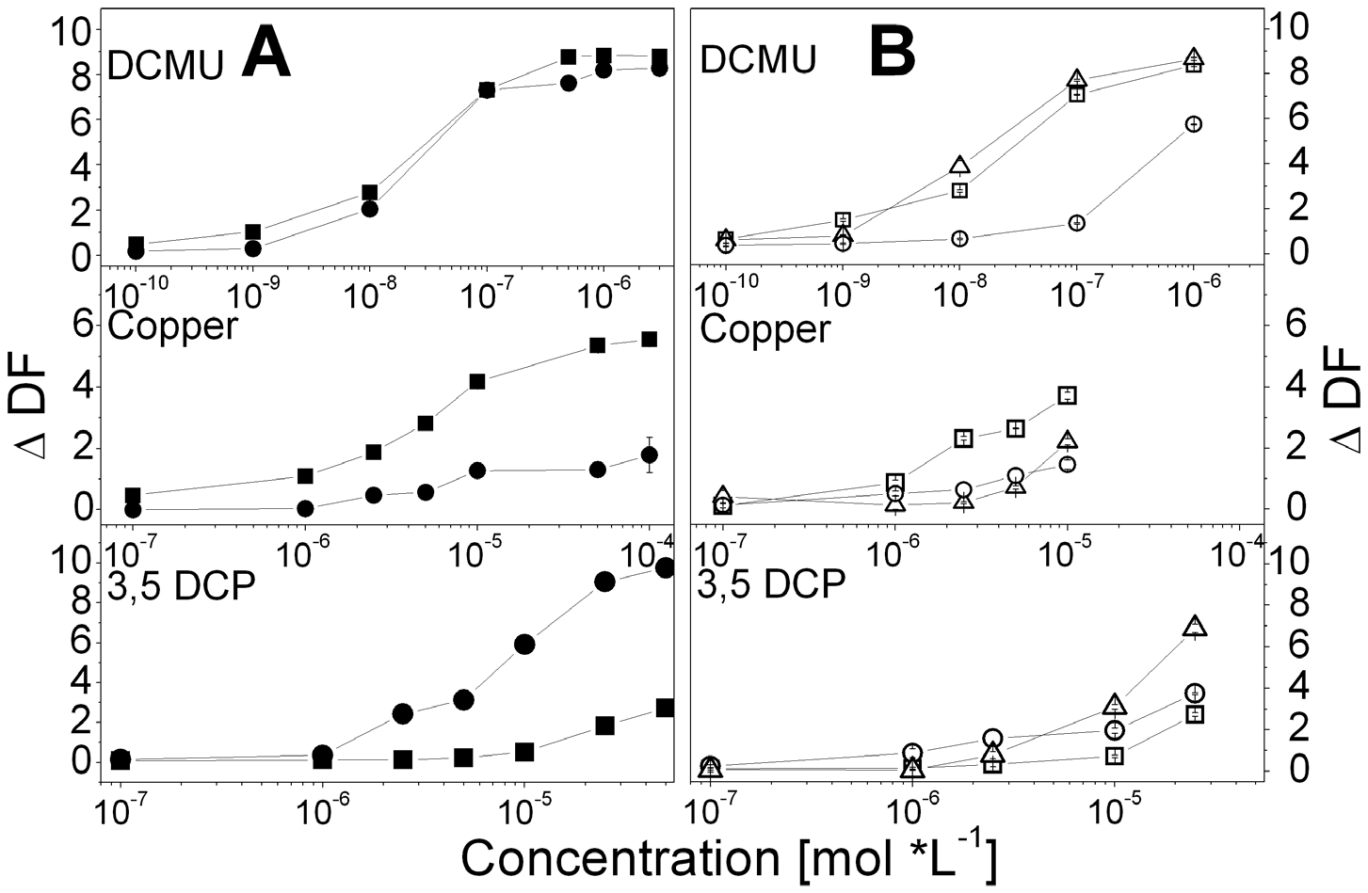
Dose response curves of ΔDF. Dose response curves of ΔDF to all three tested chemicals (DCMU, copper and 3,5 DCP) for A) *M. aeruginosa* HUB018 (squares) and *D. subspicatus* (circles); and B) *M. aeruginosa* HUBW333 (open square), *A. flos-aquae* (open triangle up) and *S. obliquus* (open circle).

All tested phytoplankton species showed a measureable ΔDF response to the tested chemicals. Cyanobacteria and green algae were highly sensitive against DCMU, but differed strongly in their sensitivity towards copper and 3,5 DCP (Fig. 3A and 3 B).

Both *Microcystis* strains showed a higher copper sensitivity than *A. flos-aquae*, *D. subspicatus* and *S. obliquus* (p<0.001 for all), whose sensitivity did not differ significantly from each other. Whereas *A. flos-aquae* was affected most by low DCMU concentrations (t-test of β, df = 22, p<0.05), *S. obliquus* was the most DCMU resistant species, the other tested strains did not differ significantly in their sensitivity. In contrast, *D. subspicatus* was influenced the strongest by low concentrations of 3,5 DCP (t-test of β, df = 24, p<0.05), followed by *S. obliquus* and *A. flos-aquae*. However, both *Microcystis* strains were significantly less affected by 3,5 DCP.

EC_50_ values were calculated for DCMU: 4.8*10^−8 ^M (HUB018) and 6.2*10^−8^ M (*D. subspicatus*), 5*10^−6^ M for copper (HUB018) and 8.1*10^−6^ M for 3,5 DCP (*D. subspicatus*). For all other tested strains and chemicals, EC_50_ values could not be calculated because saturation could not be reached.

The degree of inhibition detected by our DF-based biotest was compared with that of algal growth inhibition tests in a series of growth inhibition experiments, which were performed in parallel to DF-assays. For this comparison we had chosen *Microcystis* strain HUB018 and *D. subspicatus* because of their relatively high sensitivity to copper and 3,5 DCP, respectively (see dose-response curves in Figs. 3).

**Figure 4:**
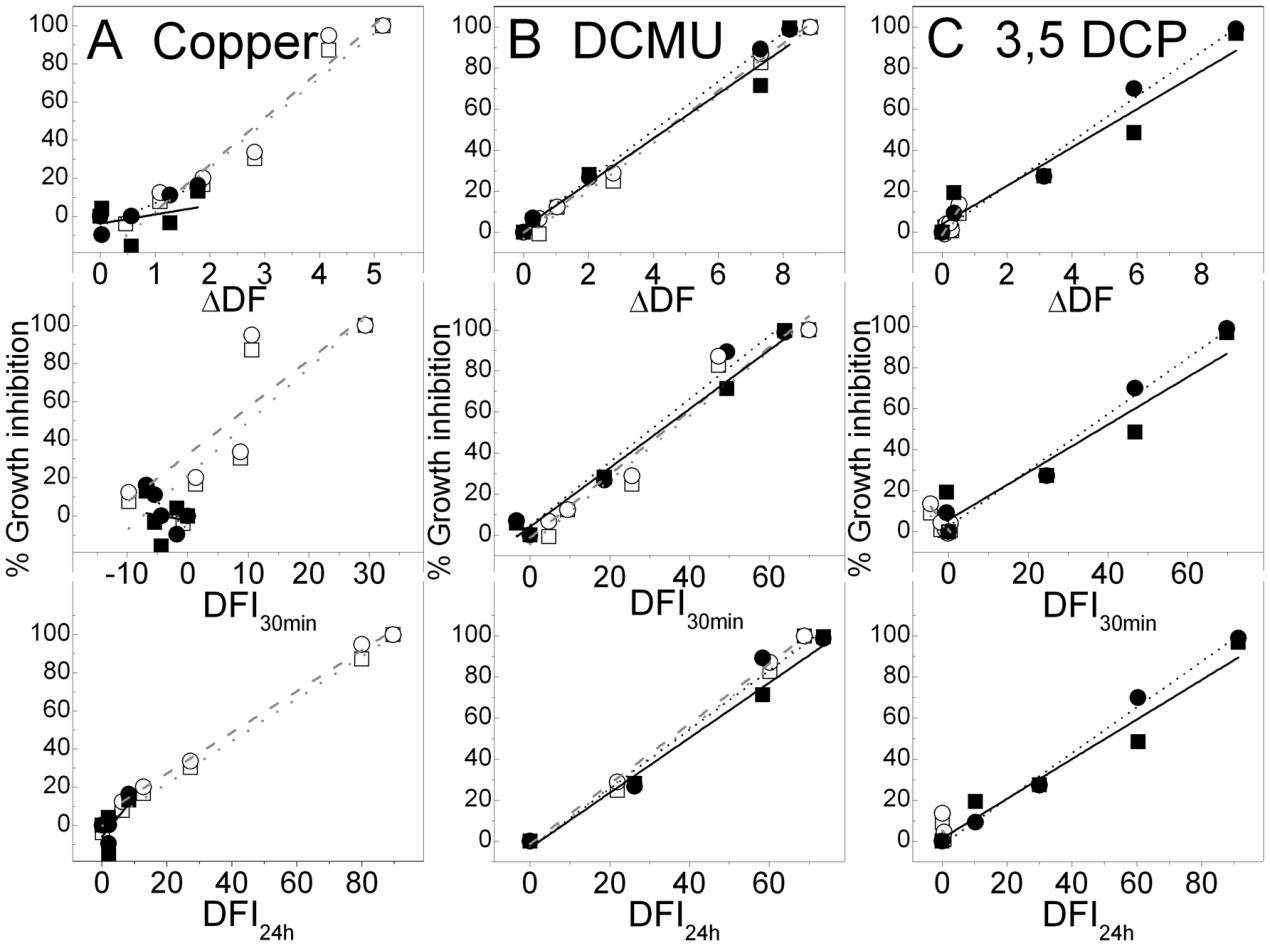
Correlation of DF parameters and growth. Correlation between the DF parameters ΔDF, DFI_30min_, DFI_24h_ with growth inhibition at day 3 (squares) and day 9 (circles) for *M. aeruginosa* (opened symbols) and *D. subspicatus* (filled symbols) as well as linear fits for *M. aeruginosa* (grey, dotted day 3, dashed day 9) and *D. subspicatus* (black, solid day 3, short dashed day 9) ) for the tested chemicals A) copper, B) DCMU, C) 3,5 DCP.

Due to their sensitivity to the added chemicals (*M. aeruginosa* HUB018 to DCMU and copper, *D. subspicatus* to DCMU and 3,5 DCP), both tested algae showed a clear relationship between ΔDF as well as DFI_30min_ and DFI_24h_ and the growth inhibition at day 3 and 9, respectively (with R^2^ values >0.9 for the linear regression; Fig. 4). There was one exception for *M. aeruginosa* tested against copper using the DFI_30min_ (R^2^>0.71). Small effects on DF and growth could be either seen for 3,5 DCP added to *M. aeruginosa* and for copper added to *D. subspicatus* (Fig. 4) caused a weaker relation. Especially, the correlation between the DF parameters and growth inhibition on day 3 were small (R^2^ = 0.12 for *D. subspicatus*, ΔDF of copper; R^2^ = 0.07, DFI_24h_ HUB018, 3,5 DCP), but still a high correlation between ΔDF and growth inhibition was reached for *D. subspicatus* at day 9 (R^2^ of 0.87, ΔDF of copper). Our results suggest that ΔDF based on changes in the shape of the DF decay kinetic yield similarly good results as changes of the DF integral even after 24 h.

## Discussion

The main goal of the present study was to confirm the suitability of a DF-based *in vivo* approach as a rapid and sensitive toxicity test for potential pollutants with cyanobacteria and green algae as indicator organisms. To achieve this goal, we focused on quantifying changes in the DF-decay curve when comparing to the respective controls. Measuring DF does not require any preparation of the algae and toxicity effects of the tested chemicals, even in low concentrations, could be obtained after 20 min of incubation. Our results indicate that ΔDF can be used as a rapid, reliable and sensitive phytoplankton toxicity test.

DF as a parameter to investigate toxicity effects on algae was used in the past for green algae in laboratory studies [18], [21], [39], but also the implementation in continuous biomonitoring stations of surface waters was suggested [40]. Further applications of DF provide a reliable tool to investigate the phytoplankton community in lakes [41], even over the seasonal aspect and vertical distribution [42] as well as online monitoring [43]. Similar findings have been obtained also for streams [44], [45], [46].

In the present study, the most obvious effect occurred with cyanobacteria, after addition of the potential pollutants. All three tested species displayed a prominent transient peak at about 25 to 35 sec (Fig. 2), whereas both green algae (*D. subspicatus* and *S. obliquus*) did not. In particular, changes in the transient peak, following the addition of toxin, render cyanobacteria promising candidates for our DF approach.

The occurrence of a transient peak has been described as the PSI involvement in DF decay kinetics after irradiation with long-wave light (650–700 nm) [20], [47], [48]. However, the spectrum of the red light LED used in our DF instrument reaches its maximum at 654 nm, thus both PS are involved in the electron transport.

A more plausible explanation for this phenomenon, which is typical for cyanobacteria, is their imbalance between both photosystems. While most photoautotrophs exhibit a PSI:PSII ratio of 1, cyanobacteria exhibit a PSI:PSII ratio of up to 3 [49]. A possible reason is the involvement of their PS-ETC’s enzymes in the respiratory ETC. Electrons of the respiratory ETC enter the PS-ETC at the Plastoquinone pool and leave it after the transition through the cytc553/Plastocyanin [50], [51]. Confirmation of the hypothesis that the observed transient DF peak is also related to respiration sustains the applicability of cyanobacteria in toxin tests as it expands the sensitivity of the test organisms to respiratory toxins.

None of the species showed an overall highest sensitivity to all tested chemicals. This finding is supported by studies, which have used growth inhibition tests to compare the sensitivity in phytoplankton species, differing in their cell-wall structure or enzyme activities [52]–[54]. Wängberg & Blanck [55] also showed that algal phylogeny can be relevant for the measured sensitivity towards toxicants.

Among the chemicals tested, the herbicide DCMU drastically steepened the DF decay kinetic during the first seconds, which can be explained by the specific action of DCMU. DCMU blocks the electron transport between PSII and the Plastoquinone pool, therefore only PS680^+^ and Q_A_^-^ molecules contribute to the DF signal. Our measured EC_50_ values for DCMU (4.8*10^−8^ M for HUB018 and 6.2*10^−8 ^M for *D. subspicatus*) are in the range for DFI_30min_ (8.58*10^−8^) and DFI_24h_ (4.29*10^−8 ^M) measured by Berden-Zrimec et al. [14]. Results obtained at low DCMU concentrations (Fig. 3), indicate that cyanobacteria are slightly more sensitive against DCMU than green algae.

Copper, as a necessary trace metal in cell metabolism, has the potential to disturb the photosynthetic electron transport at high concentrations [26] by blocking the enzymes of the PS’ dark reaction. Further, it can also cause lipid peroxidation due to Fenton reactions leading to an intracellular increase of reactive oxygen species [24], [26], [56]–[58]. Both *M. aeruginosa* strains revealed a higher sensitivity to copper, e.g. HUB018 showed an effect at already 10^−7 ^M (EC_50_ = 5*10^−6 ^M), whereas both green algae were unaffected by copper up to 2.5*10^−6^ M. In parallel to our study of *M. aeruginosa*, Rojičková & Maršálek [54] showed that the cyanobacterium *Synechococcus* sp. exhibited higher sensitivity to copper than their investigated green algae. In contrast to our findings, however, Berden-Zrimec et al. [14] found lower EC_50_ values for *D. subspicatus* (EC_50DFI_  = 3.8*10^−6 ^M), possibly caused by the continuous illumination during the 24 hour or 72 h incubation. The prolonged period of illumination potentially leads to higher production of reactive oxygen species catalyzed by copper in the Fenton or Haber-Weis reaction [59].

In contrast to copper and DCMU, the organic and more volatile 3,5 DCP, a reference substance used in OECD guideline 201 and 221 [22], [60], revealed stronger effects on green algae than on cyanobacteria. Effects of dichlorophenols on eukaryotic algae have been described earlier [14], [17], [61]. While Berden-Zrimec [14] reported an EC_50DFI24h_ of 1.38 µg L^−1^ for the green algae *D. subspicatus* (about 8.4*10^−6 ^M), Shao et al. [62] found an EC_50_ value of 23.39 mg L^−1^ (1.4*10^−4^ M) by using a luciferase marked cyanobacterium *Synechocystis* sp. indicating that cyanobacteria are less sensitive, a notion that validates our findings. Comparison of EC values must always be considered carefully, because of differences in endpoint parameters (growth, fluorescence) as well as incubation time with the tested toxin. In our study, we successfully used the parameter ΔDF taking into account changes in shape of the decay kinetic. After 20 min, all our investigated strains showed measurable effects on ΔDF, whereas it took Berden-Zrimec et al. [14] 30 min after adding 3,5 DCP to observe a decrease in the DF integral of *D. subspicatus*. However, the same authors did not find a significant effect after adding DCMU or potassium dichromate.

Furthermore, duration of an experiment can be crucial for comparing short time DF with growth inhibition tests of *M. aeruginosa* HUB018 and *D. subspicatus*. While short- term incubations only account for acute toxicity reflected by the investigated parameters, growth inhibition experiments spanning over several generations consider chronic, accumulative or mutative effects of toxins. In our study, *M. aeruginosa* HUB018 and *D. subspicatus* showed a close correlation between ΔDF and growth inhibition, except for small, measurable toxicity effects (small changes in DF and low growth inhibition over the entire tested concentration spectra). Similar to other studies [52], [54], [63], no overall sensitive species could be detected, but it will be an exciting challenge to test the DF response to a much wider range of pollutants for both phytoplankton groups, in particular effects caused by respiratory toxins on the DF decay of *Microcystis* sp. Our results indicate that our DF approach is a promising tool for investigating physiological alterations caused by chemicals and pollutants, not only in green algae as has been shown earlier [14], [17], but also for cyanobacteria. Furthermore, our DF test introduced in this study can easily be modified for on-line monitoring, where rapid and sensitive parameters are required to obtain a fast algal response towards a potential contamination [40]. Cyanobacteria that often dominate natural phytoplankton communities, should be included in such monitoring systems. It will be an exciting challenge to test the DF response to a much wider range of pollutants for both phytoplankton groups, in particular effects caused by respiratory toxins on the DF decay of *Microcystis* sp.

### Conclusions

Application of our DF approach to testing toxicity in cyanobacteria provided a reliable, rapid, and sensitive tool, which yields similar results as the applied growth inhibition tests. Since the DF approach is an *in vivo* test, it does not require time-consuming preparations and allows for automation for high frequency measurements. The sensitivity of cyanobacteria and green algae differs significantly to each tested chemicals. While green algae are commonly used in standard toxicity tests and biomonitoring, cyanobacteria, often dominating phytoplankton communities, have largely been neglected as test organisms for reliably monitoring of environmental disturbances by toxic substances. Adapting our DF approach to cyanobacteria for online monitoring seems to be promising for monitoring complex, natural phytoplankton communities.
